# Heterogeneous Fenton-like CuO-CoO_x_/SBA-15 catalyst for organic pollutant degradation: synthesis, performance, and mechanism

**DOI:** 10.3389/fchem.2025.1552002

**Published:** 2025-02-19

**Authors:** Jinwei Li, Yifei Wei, Qiang Liu, Huanhuan Guan, Chengchun Jiang, Xiaohui Sun

**Affiliations:** ^1^ School of Material and Environmental Engineering, Shenzhen Polytechnic University, Shenzhen, Guangdong, China; ^2^ School of Municipal and Environmental Engineering, Shenyang Jianzhu University, Shenyang, Liaoning, China; ^3^ College of Civil and Transportation Engineering, Shenzhen University, Shenzhen, China

**Keywords:** CuO-CoOx/SBA-15, Fenton-like system, bimetallic oxides, synergistic effect, organic pollutant degradation

## Abstract

In this study, CuO-CoO_x_/SBA-15 catalysts were successfully synthesized via ultrasonic impregnation, and their performance in degrading nitrobenzene within a Fenton-like system was investigated. The catalyst materials were characterized using X-ray diffraction (XRD), X-ray photoelectron spectroscopy (XPS), inductively coupled plasma mass spectrometry (ICP-MS), scanning electron microscopy (SEM), transmission electron microscope(TEM) and energy-dispersive X-ray spectroscopy (EDS). The CuO-CoO_x_/SBA-15 catalysts featured well-distributed CuO-CoO_x_ nanoparticles within the mesoporous SBA-15 support. Compared to CuO/SBA-15 and Co_3_O_4_/SBA-15 catalysts with similar microstructures, the CuO-CoO_x_/SBA-15 catalysts exhibited Cu-Co dual active centers and a higher abundance of redox-active sites. During catalytic degradation, H_2_O_2_ was continuously activated on the catalyst surface through efficient Cu^+^/Cu^2+^ and Co^2+^/Co^3+^ redox cycles. The experimental conditions (initial pH, catalyst dosage, and H_2_O_2_ dosage) were optimized, resulting in 99% nitrobenzene removal over a wide pH range (3.0–9.0). The primary mechanisms for the oxidation and subsequent removal of nitrobenzene in the CuO-CoO_x_/SBA-15-H_2_O_2_ system were identified as reactions with hydroxyl radicals (·OH).

## Graphical Abstract

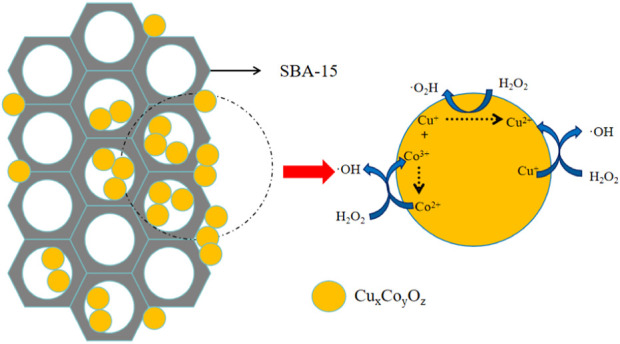

## Highlights

• CuO-CoO_x_/SBA-15 catalyst synthesized using ultrasonic impregnation method.
• Contaminants effectively degraded across a wide pH range (3.0–9.0).
• Cu-Co dual active centers drive the continuous activation of H_2_O_2_.
• Nitrobenzene degradation driven by ·OH in the CuO-CoO_x_/SBA-15-H_2_O_2_ system.

## 1 Introduction

Advanced oxidation processes (AOPs) have gained significant attention in wastewater treatment due to their exceptional effectiveness in rapidly and efficiently removing pollutants. These processes rely on highly reactive free radicals, such as hydroxyl radicals (Silveira et al., 2015; Zhu et al., 2019). Among the various AOPs, the Fenton reaction—where Fe^2+^ activates H_2_O_2_—remains the most widely studied. However, this homogeneous reaction typically operates within a low pH range (2.0–4.0) and produces a substantial amount of iron sludge (Blanco et al., 2016). As a result, heterogeneous catalysts have been explored as an alternative, leading to the development of heterogeneous Fenton-like systems that offer several advantages over traditional homogeneous Fenton reaction (Lei et al., 2015; Nidheesh, 2015).

Heterogeneous Fenton-like pollutant degradation systems primarily utilize transition metal-based catalysts (Bokare and Choi, 2014; Anipsitakis and Dionysiou, 2004) due to their excellent catalytic activity, low cost, widespread availability, and abundant reserves. In recent decades, the development of ultra-efficient heterogeneous Co- and Cu-based catalysts has garnered significant attention, driven by the broad pH tolerance and remarkable catalytic activity of these metals (Zhao Y. B. et al., 2023; Yu et al., 2019; Zhang et al., 2020). When employed as catalysts in Fenton-like reactions, nano Cu-Co bimetallic oxides have demonstrated high efficiency in degrading pollutants such as nitrobenzene and humic acid in water (Tan et al., 2024; Li et al., 2021). These metals can catalyze the generation of ⋅OH from H_2_O_2_ through the conversion between their different valence states, behaving similarly to Fe in Fenton-like reactions. At the same time, many studies suggest that the synergistic effect between different metals can accelerate electron transfer and the redox cycle of metal ions (Hammouda et al., 2017). More importantly, the uniform electronic distribution induced by oxygen vacancies in catalysts prepared with two different metals further promotes the adsorption and activation of reactant species on Cu-Co bimetallic oxides (Yu et al., 2019; Zhao Z. et al., 2023). However, despite the high performance of some bimetallic catalysts, bimetallic oxide nanoparticles often exhibit low crystallinity and significant particle size variations. Additionally, van der Waals forces and electrostatic interactions can lead to the agglomeration of these nanoparticles into larger particles, which negatively impacts their catalytic or adsorptive performance. Due to their small size, further challenges, such as difficulties in separation and issues with diffusion, hinder the widespread practical use of nanoparticle catalysts in wastewater treatment. Therefore, developing methods to prepare nanoparticles on an appropriate support matrix to achieve good particle dispersion and facilitate easy separation from treated wastewater, while maintaining excellent Fenton-like catalytic activity, would be highly desirable.

To address the aforementioned issues, some researchers have immobilized bimetallic oxides onto various supports, such as metal oxide (Sehati and Entezari, 2017), clay, zeolites, and carbon-based materials (Liu et al., 2019; Du et al., 2019; Prathap et al., 2012). The use of these supports helps prevent metal ion leaching and provides a larger specific surface area (Galarneau et al., 2007), thereby enhancing catalyst stability and promoting reactant adsorption. The performance of heterogeneous catalytic materials is closely linked to their active centers, which are directly influenced by their nanomorphology. A well-designed heterogeneous catalyst features numerous highly dispersed active centers on a porous support. As a result, materials with high mesoporosity have garnered significant interest. However, the nanochannels in these mesoporous supports can become blocked, leading to reduced support utilization and diminished performance.

In this study, an innovative ultrasonic impregnation method was employed to synthesize heterogeneous catalyst materials with novel nanoarchitectures within the confined spaces of ordered mesoporous hosts. First, an alcohol precursor solution was prepared, and the mesoporous host was added to this solution. Thorough impregnation of the host’s pore channels with the precursors was ensured through continuous stirring and ultrasonication. Subsequently, a heating step was applied to decompose the precursors within the confined pore spaces. This strategy was used to produce well-dispersed and uniform metal oxide nanoparticles within the nanochannels of the host material.

Ultrasonic impregnation was then used to prepare a series of CuO-CoO_x_/SBA-15 catalysts. Mesoporous SBA-15 (Santa Barbara Amorphous-15), with its large surface area, excellent structural stability in water (Galarneau et al., 2007), highly ordered pores and channels, and narrow pore size distribution, is an ideal support material for catalysts. The suitable pore sizes range (5–8 nm or more) provided by SBA-15 creates a confined microenvironment that facilitates the growth of nanoparticles. Nitrobenzene, a common organic intermediate and emerging environmental contaminant, was chosen as the target pollutant to assess the catalytic degradation efficiency of CuO-CoO_x_/SBA-15 as a Fenton-like catalyst. A series of characterization techniques were employed to study the chemical composition and physical structure of the prepared catalysts. The synergistic effect of the copper and cobalt oxides on catalytic nitrobenzene degradation was evaluated, and the experimental degradation parameters (pH, catalyst dosage, and H_2_O_2_ dosage) were optimized to enhance nitrobenzene removal. Finally, a potential H_2_O_2_ activation mechanism over CuO-CoO_x_/SBA-15 was proposed. Overall, this study provides a straightforward preparation strategy for obtaining highly active Fenton-like catalysts.

## 2 Materials and methods

### 2.1 Materials

The following reagents were obtained from Aladdin (Shanghai, China): nitrobenzene (C_6_H_5_NO_2_, NB, 99.0%), tetraethylorthosilicate (Si(OC_2_H_5_)_4_, TEOS, 98%), ethanol (C_2_H_5_OH, 99.8%), and polyethylene oxide-polypropylene oxide-polyethylene (P123, MW = 5800). Cu(NO_3_)_2_·4H_2_O and Co(NO_3_)_3_·6H_2_O were purchased from Tianjin Guangfu Fine Chemical Co. (China). H_2_O_2_ (30%) was obtained from Beijing Chemical (China). HPLC-grade methanol was purchased from Fisher Scientific (United States). H_2_SO_4_ (0.1 M) and NaOH (0.1 M) were used to adjust the pH of the reaction solution.

### 2.2 Synthesis of catalysts

SBA-15 was synthesized hydrothermally (Liu et al., 2019). In detail, 4.0 g of P123 was added to 120 mL of an aqueous HCl solution (100 mL water +20 mL HCl). This solution was then heated to 40°C and stirred until it became clear. Next, 8.6 mL of TEOS was slowly added to the solution, which was stirred for 24 h to obtain a sol. The sol was hydrothermally reacted in a polytetrafluoroethylene-lined autoclave reactor for 48 h at 100°C. The resulting solid product was cooled, washed with deionized water until a neutral pH was achieved, and then dried. Finally, the product was calcined for 6 h at 500°C to obtain pure SBA-15.

An ultrasonic impregnation method was used to prepare CuO-CoO_x_/SBA-15. Specifically, 0.5 g Cu(NO_3_)_2_·3H_2_O and 0.122 g Co(NO_3_)_3_·6H_2_O were dissolved in 5 mL ethanol. This ethanol solution was gradually added to 0.6 g SBA-15, and the mixture was stirred for 3 h to form a paste. Ultrasonic impregnation was then performed for 30 min with an ultrasonic power of 0.5 W/cm^3^, followed by calcination for 5 h at 300°C in a muffle furnace to obtain CuO-CoO_x_/SBA-15. A series of CuO-CoO_x_/SBA-15 catalysts were prepared by varying the total metal content, Cu/Co molar ratio, and calcination temperature. Samples labeled M_1_, M_2_, M_3_, M_4_, and M_5_ were prepared with total metal oxide contents of 1.08 wt%, 2.3 wt%, 3.4 wt%, 4.9 wt%, and 5.1 wt%, as determined by inductively coupled plasma mass spectrometry (ICP-MS). The Cu/Co molar ratio of in all samples was 1:2, unless otherwise specified.

### 2.3 Catalyst characterization

N_2_ adsorption-desorption isotherms were acquired with a TRISTAR II 3020 (Micromeritics Instrument Corporation, United States), and the Barrett-Joyner-Hallender (BJH) pore size distributions were determined from the desorption branch. Specific surface areas were calculated using the Brunauer-Emmett-Teller (BET) theory. X-ray diffraction (XRD) analysis was conducted with a Bruker AXS D8 system, using Cu Ka radiation (λ = 1.540598 Å) in the 2θ range of 10°–80° to assess the crystal structures. X-ray photoelectron spectroscopy (XPS) was performed with an ESCALAB 250Xi (Thermo Electron Corporation, United States) using Al Kα radiation (1,486.6 eV). The C 1s peak at 284.6 eV was used as a reference for peak calibration. Field emission scanning electron microscopy (FE-SEM), transmission electron microscopy (TEM) and scanning transmission electron microscopy coupled with energy-dispersive X-ray spectroscopy (STEM-EDS) (JEM-3200FS) were employed to examine the morphology, particle size, phases, and surface elemental distributions of the samples. Inductively coupled plasma mass spectrometry (ICP-MS, NexION 2000, PerkinElmer) was used to quantify the metal content in each sample.

### 2.4 Fenton-like catalytic degradation testing

The catalytic degradation of nitrobenzene (NB) was performed at room temperature. An NB solution with a concentration of 50 mmol/L was first prepared and adjusted to the desired pH. About 0.2 g of catalyst was then added to 100 mL of this solution. Before adding H_2_O_2_, the mixture was magnetically stirred for 30 min in the dark to ensure adsorption equilibrium. Afterward, a specified amount of 30.0 wt% H_2_O_2_ was added under continuous stirring to initiate the Fenton-like degradation reaction. At regular intervals of 15 min, small samples of the reaction mixture were withdrawn with a syringe and filtered through a 0.22 μm membrane filter. High-performance liquid chromatography (HPLC) was employed to analyze each filtered sample, and the NB concentration was determined using a Waters e2695 system with a C18 column. The chromatography analysis was performed with a mobile phase of 70% methanol and 30% formic acid solution (0.1 vol%), a flow rate of 1 mL/min, an injection volume of 50 μL, and a detection wavelength of 265 nm.

## 3 Results and discussion

### 3.1 Validation of CuO-CoO_x_/SBA-15-H_2_O_2_ system for nitrobenzene decomposition

The NB removal efficiencies of H_2_O_2_ (100 mmol/L), CuO-CoO_x_/SBA-15 (2.0 g/L), and CuO-CoO_x_/SBA-15-H_2_O_2_ (2.0 g/L catalyst, 100 mmol/L H_2_O_2_) systems are shown in Supplementary Figure S1. Less than 20% NB removal was achieved by H_2_O_2_ or CuO-CoO_x_/SBA-15 alone, demonstrating that neither of these reagents alone could effectively degrade NB. However, the combination of both H_2_O_2_ and CuO-CoO_x_/SBA-15 resulted in an NB degradation efficiency of approximately 80.0% within 60 min. This demonstrates that CuO-CoO_x_/SBA-15 can effectively catalyze H_2_O_2_ for the removal of NB, which is consistent with our previous report (Li et al., 2021).

### 3.2 Influence of calcination temperature on the properties of CuO-CoO_x_/SBA-15 and NB degradation performance

Different CuO-CoO_x_/SBA-15 catalysts were prepared by calcining at temperatures of 300°C, 400°C, and 500°C.Both Pure SBA-15 and the CuO-CoO_x_/SBA-15 catalysts exhibited typical N_2_ adsorption-desorption type-IV isotherms, each featuring H1-type hysteresis loops in the range of p/p_0_ = 0.6–0.8 (Supplementary Figure S2). This behavior is attributed to the well-defined hexagonal pore structure of pure SBA-15. The CuO-CoO_x_/SBA-15 catalysts calcined at different temperatures had similar pore volumes (approximately 0.80 cm^3^/g) and pore sizes (about 5.5 nm). The specific surface areas of the calcined CuO-CoO_x_/SBA-15 catalysts (Table 1) were slightly lower than that of pure SBA-15 but significantly higher than those of CuO-CoO_x_ and other CuO-containing catalyst materials, such as CuO/rGO (56.1 m^2^/g) (Du et al., 2019) and CuO/Ti_6_O_13_ (6.93 m^2^/g) (Sehati and Entezari, 2017). Higher specific surface areas facilitate faster electron transfer (Prathap et al., 2012), thereby enhancing catalytic activity. These results suggest that the ultrasonic impregnation method enables a uniform distribution of CuO-CoO_x_ on the SBA-15 surface, which likely altered the surface structure of the catalyst and facilitated the catalytic reaction. The observed decrease in pore diameters and volumes of the catalysts after ultrasonic impregnation further confirms the successful loading of CuO-CoO_x_ onto SBA-15 support material.

**TABLE 1:** BET specific surface area, pore volume, and pore size of CuO-CoOx/SBA-15 catalysts prepared at different calcination temperatures, Cu/Co molar ratios, and metal oxide loadings.

		S_BET_ (m^2^/g)	Vpore (cm³/g)	Dp (nm)
	SBA-15	674	0.96	5.7
calcination temperature	300°C	588	0.86	5.5
	400°C	535	0.77	5.5
	500°C	517	0.77	5.7
different Cu/Co molar ratios	CuO/SBA-15	614	0.83	5.3
	CoO_2_/SBA-15	585	0.82	5.3
	Cu_2_CoO_x_/SBA-15	600	0.82	5.2
	CuCoO_x_/SBA-15	588	0.79	5.3
	CuCo_2_O_x_/SBA-15	588	0.86	5.5
	CuCo_3_O_x_/SBA-15	555	0.83	5.8
metal oxide loading content (Cu/Co = 1:3)	M_1_	602	0.96	6.2
	M_2_	574	0.92	6.1
	M_3_	589	0.86	5.3
	M_4_	588	0.86	5.5
	M_5_	484	0.74	5.3

The phases of CuO-CoO_x_/SBA-15 and pure SBA-15 were evaluated by XRD, as shown in Supplementary Figure S3. All samples exhibited a broad peak in the 2θ range of 20°–23° (Supplementary Figure S3a), confirming the formation of SBA-15. According to Thahir et al. (Thahir et al., 2019), this peak is attributed to the [100] planar orientation of SBA-15. The samples calcined at 300°C and 400°C did not display any other diffraction peaks, indicating that metal oxide crystals did not form at these calcination temperatures. However, when the calcination temperature was increased to 500°C, overlapping peaks ascribed to Cu_2_O and Co_3_O_4_ were observed at 38°, indicating that higher calcination temperatures promote the formation of metal oxide particles. The absence of other diffraction peaks indicated that no other phases were generated or that they were present in negligible amounts.

**FIGURE 1:**
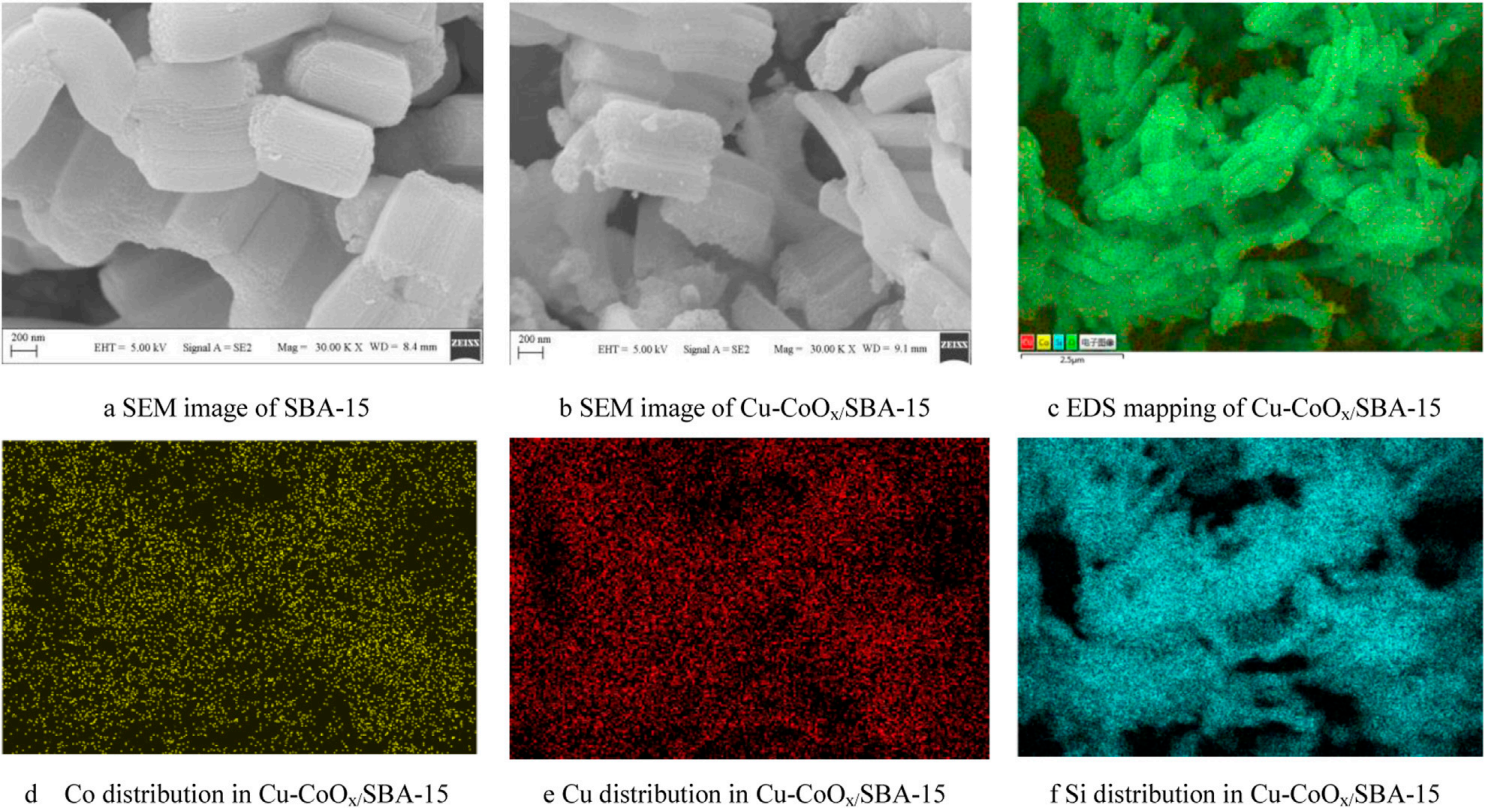
SEM images of SBA-15 **(A)** and CuO-CoO_x_/SBA-15 **(B)**; EDS analysis showing the distributions of Co, Cu, and Si in CuO-CoO_x_/SBA-15 **(C–F)**.

The morphologies of SBA-15 and CuO-CoO_x_/SBA-15 were analyzed by SEM and TEM, as shown in Figure 1and Supplementary Figure S5. The SEM image of SBA-15 (Figure 1A) revealed long cylindrical rod-shaped particles, consistent with previous studies on SBA-15 (Kumaravel et al., 2020). In contrast, SEM analysis of CuO-CoO_x_/SBA-15 (Figure 1B) demonstrated that CuO-CoO_x_ was uniformly distributed throughout the SBA-15 structure. TEM analysis of CuO-CoO_x_/SBA-15(Supplementary Figure S5) shown that the CuO-CoO_x_ particles were smaller than 80 nm, which is expected to enhance the degradation performance. This observation confirms that SBA-15 effectively control the shape and size of CuO-CoO_x_ nanoparticles. EDS analysis (Figures 1D–F) and elemental mapping (Figure 1C) further validated the uniform dispersion of Co, Cu, and Si within the CuO-CoO_x_/SBA-15 catalyst. The even distribution of elements and the uniform particle size of CuO-CoO_x_ can be attributed to the cavitation effect induced by ultrasonication, which created a highly intense environment during catalyst preparation.

**FIGURE 2:**
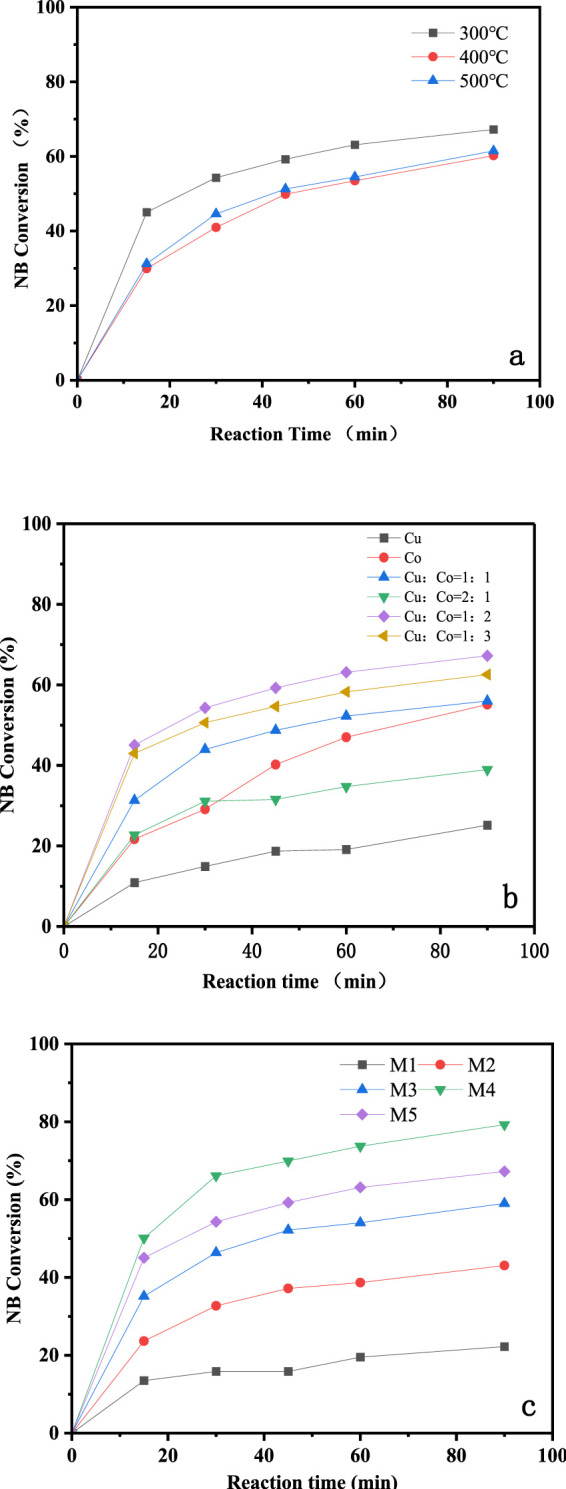
Degradation efficiencies of NB by CuO-CoO_x_/SBA-15 catalysts prepared using different calcination temperatures **(A)**, Cu/Co molar ratios **(B)**, and metal oxide content **(C)**.

The CuO-CoO_x_/SBA-15 catalysts, calcined at different temperatures, were evaluated for NB degradation in the presence of H_2_O_2_ under the following conditions: 25°C reaction temperature, 2.0 g/L catalyst dosage, 100 mmol/L H_2_O_2_ dosage, 50 mmol/L NB concentration, initial pH of 7.5, and a reaction time of 90 min, as shown in Figure 2A. After calcination at 300°C, 400°C, and 500°C, the CuO-CoO_x_/SBA-15 catalysts achieved degradation efficiencies of 67.24%, 50.21%, and 51.49%, respectively. The characterization results of the specific surface area (as shown in Table 1) indicate that as the calcination temperature increases from 300°C to 500°C, the specific surface area of the obtained catalysts gradually decreases from 588 m^2^/g to 517 m^2^/g, and the catalytic activity of the corresponding sample also gradually decreases. The lower degradation efficiencies observed at higher calcination temperatures are consistent with the poor crystal shape, rough surface, low dispersibility, and lack of regularity observed at lower temperatures. Based on these findings, 300 °C was determined to be the optimal calcination temperature.

### 3.3 Influence of Cu/Co molar ratio on CuO-CoO_x_/SBA-15 properties and NB degradation efficiency

Heterogeneous bimetallic oxide catalysts (e.g., Cu-Mn) can be effectively employed for the removal of target contaminants in Fenton-like degradation systems. Zhang et al. (2020), Qin et al. (2020) reported that the combined advantages of each catalytic component in bimetallic oxide catalysts can be effectively leveraged, resulting in synergistically enhanced reaction performance compared to pure transition metal oxide catalysts. However, the underlying synergistic mechanisms and the individual contributions of the two metals in bimetallic oxide catalysts remain poorly understood.

In this study, to evaluate the effect of the Cu/Co metal oxide ratio on NB degradation, a series of monometallic and bimetallic oxide catalysts supported on SBA-15 were prepared. As shown in Figure 2B, the monometallic oxide catalysts exhibited low NB removal efficiencies, highlighting the synergistic effect of combining Cu and Co oxides in the Fenton-like reaction. With a fixed CuO-CoO_x_ content of 3.4 wt%, the NB removal efficiency was significantly enhanced as the cobalt content increased. The CuO-CoO_x_/SBA-15 catalyst prepared with a Cu/Co molar ratio of 1:2 demonstrated the highest NB removal efficiency.

The CuO/SBA-15 catalyst exhibited a specific surface area of 614 m^2^/g (Table 1), suggesting that ultrasonic impregnation effectively facilitated the uniform distribution of CuO on the SBA-15 surface. However, as the CoO_x_ content increased, the specific surface area decreased to 588 m^2^/g and 555 m^2^/g, respectively. The CuO-CoO_x_/SBA-15 catalysts prepared with varying CuO/CoO_x_ ratios showed similar pore volumes (0.79–0.86 cm^3^/g), although the pore size increased to 5.8 nm when the CuO/CoO_x_ ratio was 1:3. So, the effect of copper/cobalt ratio on the specific surface area of the catalyst is not significantly positively correlated with their respective degradation efficiencies.

The XRD patterns of the monometallic and bimetallic CuO-CoO_x_/SBA-15 catalysts with various Cu/Co ratios are shown in Supplementary Figure S3B. As the CoO_x_ content increased, the diffraction peak corresponding to Co_3_O_4_ became more prominent, while the diffraction peaks for CuO remained relatively unchanged. This suggests that the presence of CoO_x_ helps to disperse CuO and inhibits its aggregation, suggesting a synergistic effect between the two metal oxides.

**FIGURE 3:**
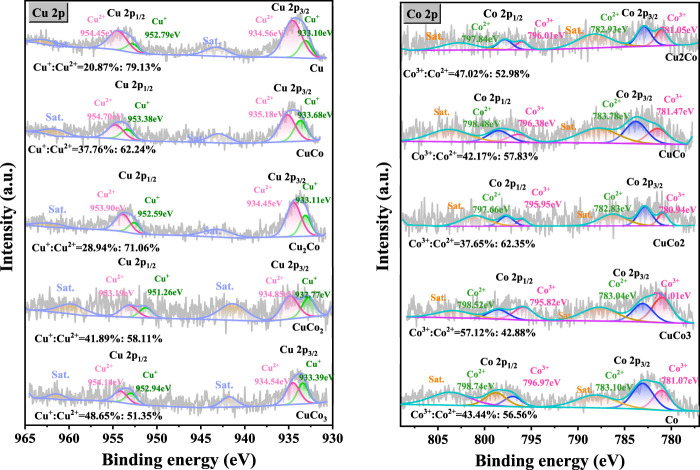
Cu 2p and Co 2p XPS spectra of CuO-CoO_x_/SBA-15 catalysts with different metal ratios.

The Cu 2p and Co 2p XPS spectra of the CuO-CoO_x_/SBA-15 catalysts prepared with different metal ratios are shown in Figure 3. The Cu 2p_3/2_ peak was deconvoluted into two distinct peaks corresponding to Cu(II) and Cu(I) at 934.56 eV and 933.10 eV, respectively (Liu et al., 2019; Lyu et al., 2015). A satellite peak for CuO was observed at 942.27 eV, approximately 9 eV higher than the main peak. The Cu(I)/Cu(II) atomic ratio, calculated from the areas of Cu(I) and Cu(II) peaks, increased with the cobalt content, with the lowest ratio (0.27:1) observed for CuO/SBA-15. This indicates a strong interaction between copper and cobalt, enhancing the redox cycle of the copper species. The Co 2p XPS spectra of the CoO_x_/SBA-15 catalyst (prepared without Cu) showed two main 2p_3/2_ peaks at 783.10 eV and 781.07 eV, corresponding to Co(II) and Co(III), respectively. Additionally, a CoO_x_ satellite peak was observed at 787.6 eV (Wang et al., 2020). Notably, the XPS spectra of the CuO-CoO_x_/SBA-15 catalysts exhibited a slight shift in binding energy. For the catalyst synthesized with a Cu/Co ratio of 1:3, the Co^3+^ peaks were located at 781.01 and 795.82 eV, the Co^2+^ peaks at 783.04 and 798.52 eV, and the satellite peaks at 787.3 and 804.3 eV. This shift in peak positions suggest a sttrong synergistic interaction between Co and Cu (Liu et al., 2019), which facilitates the Co and Cu redox processes and enhances ·OH production. The Co^2+^/Co^3+^ ratio in these catalysts varied with increasing CuO_x_ content, reaching its highest value at a Cu/Co ratio of 1:2. According to the report of Meng et al. (2024), the lower valance state of Co is closely related to the vast oxygen vacancy (Ov) in CuO-CoO_x_/SBA-15 catalysts. From Supplementary Figure S5, it can be seen that O 1s spectra are fitted into two peaks, with the peak at 530.96 eV in accordance with the lattice oxygen (O_latt_) and the peak at 531.78 eV belong to surface oxygen (O_surf_) (Meng et al., 2024; Sui et al., 2024). As is well recognized, O_surf_ is incubated by trapping of gaseous O_2_ in the oxygen vacancy (Ov) structure, which was a reactive oxygen specie to attack organics.This kind of conversion demonstrated that O_latt_ species participated in the degradation process of NB, which was consistent with the study by Sedmak et al. (2003).

### 3.4 Influence of metal oxide loading content on CuO-CoO_x_/SBA-15 properties and NB degradation efficiency

The CuO-CoO_x_/SBA-15 catalysts with higher metal oxide loading exhibited smaller specific surface areas, as shown in Table 1. This was likely due to the aggregation of CuO-CoO_x_ into larger crystalline particles. Additionally, increasing the CuO-CoO_x_ content resulted in a reduction in pore volume, which was attributed to excessive metal oxide loading blocking the pores. The XRD patterns of CuO-CoO_x_/SBA-15 catalysts prepared with varying CuO-CoO_x_ content are shown in Supplementary Figure S3C. For catalysts with CuO-CoO_x_ content of 3.4% or less, no distinct diffraction peaks for Cu_2_O and Co(OH)_2_ were observed. However, when the CuO-CoO_x_ content increased to 5.1%, clear and sharp diffraction peaks for Cu_2_O and Co_3_O_4_ appeared at 38.8° and 44.4°, indicating the formation of more crystalline Cu_2_O and Co_3_O_4_ with larger grain sizes. This phenomenon was consistent with the nitrogen adsorption-desorption analysis.

The NB degradation efficiencies of the CuO-CoO_x_/SBA-15 catalysts with varying metal oxide content are presented in Figure 2C. The catalysts with CuO-CoO_x_ contents of 1.08 wt%, 2.3 wt%, 3.4 wt%, 4.9 wt%, and 5.1 wt% achieved NB degradation efficiencies of 43.1%, 59.1%, 67.3%, 80.25%, and 16.7%, respectively. The NB degradation efficiency initially increased and then decreased with increasing CuO-CoO_x_ content, with the highest performance observed at 4.9 wt%. This enhanced efficiency can be attributed to the increased amount of active catalytic components at this loading, which facilitated the degradation reaction.

### 3.5 Optimization of operational parameters for nitrobenzene removal

To further optimize NB degradation in the CuO-CoO_x_/SBA-15-H_2_O_2_ Fenton-like system, the catalyst dosage, H_2_O_2_ dosage, and initial pH were adjusted.

**FIGURE 4:**
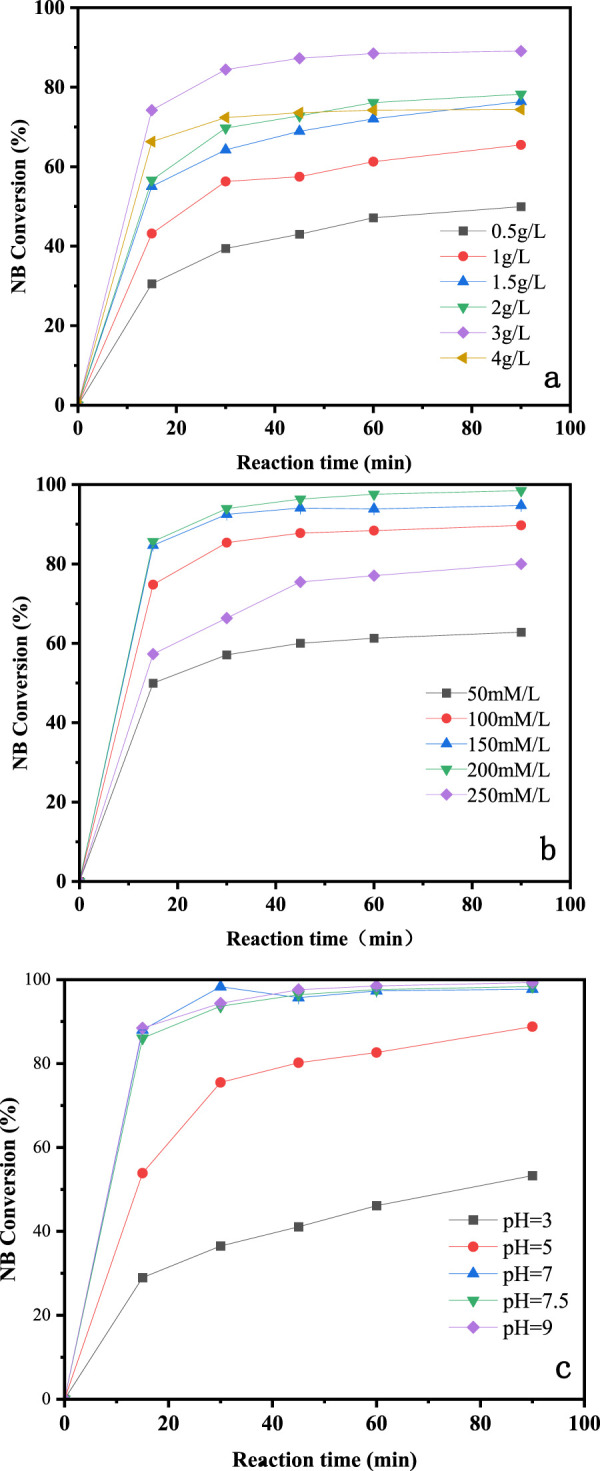
Influence of catalyst dosage **(A)**, concentration of H_2_O_2_ **(B)**, and initial pH **(C)** on degradation of NB by CuO-CoO_x_/SBA-15 in water.

The NB removal performance of CuO-CoO_x_/SBA-15 at different catalyst dosages is shown in Figure 4A. As the catalyst dosage increased from 0.5 to 3.0 g/L, the NB removal efficiency improved from 50.0% to 89.0%. This increase was attributed to a higher catalyst dosage providing more surface active sites. However, when the catalyst dosage was further increased to 4.0 g/L, no significant improvement in performance was observed. This plateau effect is likely due to the sufficient availability of active sites at the 3.0 g/L dosage. Therefore, a CuO-CoO_x_/SBA-15 dosage of 3.0 g/L was determined to be optimal for the best degradation performance.

The presence of H_2_O_2_ in the CuO-CoO_x_/SBA-15-H_2_O_2_ system resulted in the generation of ·OH, making the H_2_O_2_ dosage a key factor influencing the NB oxidation process. In this study, NB degradation was evaluated with H_2_O_2_ dosages rangin from 50 to 250 mmol/L, as shown in Figure 4B. As the H_2_O_2_ dosage increased to 200 mmol/L, the NB removal efficiency correspondingly improved, reaching 99.0%. However, further increasing the H_2_O_2_ dosage to 250 mmol/L led to a decrease in NB removal efficiency. This decline was attributed to excess H_2_O_2_ reacting with ·OH (thereby negatively affecting NB removal) or decomposing into H_2_O and O_2_, which did not contribute to NB degradation (Ghasemi et al., 2020; He et al., 2020). Based on these findings, and considering the treatment cost, the optimal H_2_O_2_ dosage was determined to be 200 mmol/L.

The degradation of organic compounds by AOPs is notably affected by pH. Conventional Fenton reactions typically operate optimally in the pH range of 2–4 (Bokare and Choi, 2014), while heterogeneous Fenton-like systems usually show broader pH tolerance (Zhang et al., 2020; He et al., 2020; Xing et al., 2008). For example, Xing et al. (2011) developed an Fe_3_O_4_/FeMnO_x_ Fenton-like catalyst that achieved excellent methylene blue (MB) removal performance in the pH range of 3.5–9.0. In this study, NB degradation was evaluated at initial pH values of 3.0, 5.0, 7.5, and 9.0, as shown in Figure 4C (reaction conditions: 25°C reaction temperature, 3.0 g/L catalyst dosage, 200 mmol/L H_2_O_2_ dosage, 50 mmol/L NB concentration, and a reaction time of 90 min). The highest NB removal efficiency of 99.0% was achieved at an initial pH of 9.0, with similar performance observed at pH 7.0 and 7.5. A pH of 5.0 resulted in slightly reduced performance, and the NB removal efficiency significantly decreased at pH 3.0. These results demonstrate that NB degradation was notably influenced by the initial pH, and the CuO-CoO_x_/SBA-15-H_2_O_2_ system was highly effective in decomposing NB under neutral or alkaline conditions.

### 3.6 Proposed mechanism of H_2_O_2_ activation by CuO-CoO_x_/SBA-15

#### 3.6.1 Identification of reactive species

**FIGURE 5:**
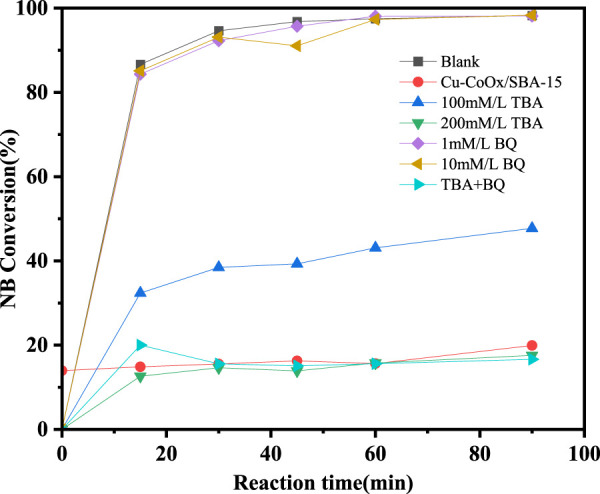
Inhibitory effects of TBA and BQ on degradation of nitrobenzene in CuO-CoO_x_/SBA-15-H_2_O_2_ system.

The reactive radicals in the CuO-CoO_x_/SBA-15-H_2_O_2_ system were identified using p-benzoquinone (BQ) and t-butanol (TBA) as quenching agents for ·O_2_^−^ and ·OH, respectively, as shown in Figure 5. In a blank experiment without any quencher, the CuO-CoO_x_/SBA-15-H_2_O_2_ system achieved 99% NB degradation after 90 min. However, the addition of TBA significantly inhibited the degradation process. Specifically, the addition of 100 mmol/L TBA reduced the NB degradation efficiency from 99% to 48.1%, and the reaction was completely inhibited with a TBA concentration of 200 mmol/L. Similar results were obtained when 200 mmol/L TBA and 10 mmol/L BQ were introduced simultaneously. Interestingly, the addition of 1 mmol/L or 10 mmol/L BQ did not hinder NB removal, suggesting that ·O_2_^−^ was not involved in the degradation process.

To further reveal the reaction mechanism, the radical species generated in the CuO-CoO_x_/SBA-15-H_2_O_2_ system were detected by performing EPR analysis with DMPO as a spin-trapping agent, as displayed in Supplementary Figure S4. The presence of ·OH was demonstrated by the DMPO-·OH adduct quartet peaks (1:2:2:1 intensity ratio, ɑ_N_ = ɑ_H_ = 14.9 G). Peaks ascribed to DMPO-·O_2_^−^ adducts were also observed. Therefore, both O_2_^−^ and ·OH existed in the CuO-CoO_x_/SBA-15-H_2_O_2_ system, and as indicated by the BQ and TBA quenching experiments, the ·OH radicals were responsible for NB degradation. It should be noted that not all of the OH generated in this system took part in NB oxidation. Instead, some of these ·OH radicals would be converted to O_2_^−^ or other species. The relative contribution of each of these reactive species will be further studied in subsequent research.

#### 3.6.2 Potential mechanism of H_2_O_2_ activated by CuO-CoO_x_/SBA-15

**FIGURE 6:**
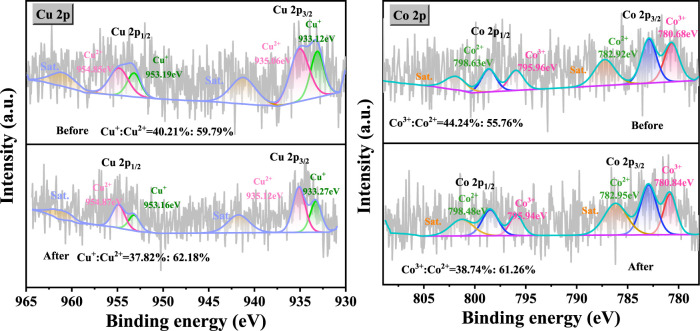
Cu 2p and Co 2p XPS spectra of CuO-CoO_x_/SBA-15 catalyst before and after NB degradation reaction.

The CuO-CoO_x_/SBA-15 catalyst surface was evaluated by XPS before and after the NB degradation reaction to further reveal the mechanism explaining its enhanced performance, as exhibited in Figure 6. The survey spectra confirmed the existence of both Co and Cu in the fresh and used catalysts as well as the absence of impurities. The Cu 2p spectrum of the fresh CuO-CoO_x_/SBA-15 catalyst contained Cu 2p_3/2_ and Cu 2p_1/2_ peaks at 934.9 and 954.4 eV (Liu et al., 2019; Wang et al., 2020), respectively. The splitting energy of 19.50 eV was close to the standard value of 19.75 eV for Cu 2p orbits, indicating that the Cu in this catalyst existed in a mixed valence state. The Cu 2p_3/2_ peak was deconvoluted into two peaks ascribed to Cu(I) and Cu(II) that were located at 933.12 and 935.06 eV, respectively. The Cu^2+^/Cu^+^ ratios of CuO-CoO_x_/SBA-15 before and after the reaction were 1.48 and 1.64, respectively. These results indicate the oxidation of Cu(I) to Cu(II), which was responsible for generating ·OH as shown in Equation 1. The Co 2p spectrum of the fresh CuO-CoO_x_/SBA-15 catalyst showed two notable peaks at 782.5 eV (Co 2p_3/2_) and 796.8 eV (Co 2p_1/2_) as well as satellite peaks at 786.6 eV and 802.5 eV (Xing et al., 2011; Ren et al., 2015; Rekha et al., 2015; Cheng et al., 2013; Yao et al., 2015). The Co 2p_3/2_ peak was deconvoluted into Co^2+^ and Co^3+^ peaks, which were respectively located at 780.68 eV and 782.92 eV. The Co^2+^/Co^3+^ ratios of the catalyst before and after the reaction were 1.26 and 1.58, respectively. This indicated that Co(II)/Co(III) oxidation played a minor role in generating ·O_2_^−^, as described by Equation 8, (Tan et al., 2024). This analysis confirms that the CuO-CoO_x_/SBA-15 catalyst was oxidized during the NB degradation reaction, which led to greater Co^3+^ and Cu^2+^ surface content. These chemical state changes are associated with the presence of H_2_O_2_. As described in Equations 1, 7, (Yao et al., 2015), the active sites on the CuO-CoO_x_/SBA-15 surface undergo oxidation from M_surf_Co^2+^ and M_surf_Cu^+^ to M_surf_Co^3+^ and M_surf_Cu^2+^. This generates oxidizing radicals (i.e., OH^−^and ·OH) that contribute to NB degradation. To complete the catalytic cycle, the M_surf_Cu^2+^ and M_surf_Co^3+^ can be reduced via a mechanism involving H_2_O_2_, as described in Equations 2–5, 8–10 (Tan et al., 2024; Guo et al., 2020). Therefore, in the presence of the Co-Cu binary oxides of CuO-CoO_x_/SBA-15, H_2_O_2_ is continuously activated and oxidizing radicals are continuously generated, promoting NB oxidation.

**FIGURE 7:**
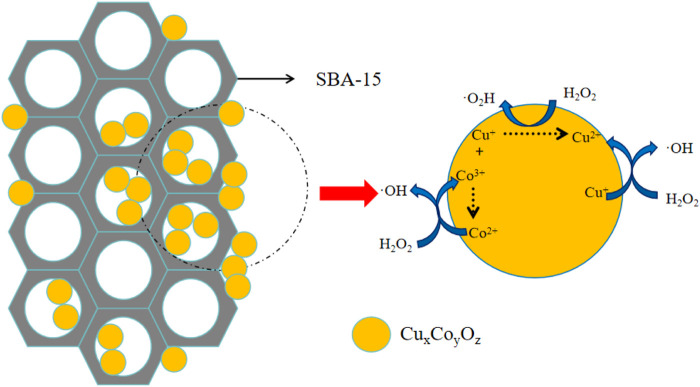
Possible mechanisms of heterogeneous Fenton-like CuO-CoO_x_/SBA-15-H_2_O_2_ catalytic system.

According to the XPS analysis, the catalytic NB degradation process involved both Co and Cu active sites. Due to its unique structure and intrinsic properties, the Co-Cu binary oxide catalyst effectively provides Co^2+^, Co^3+^, Cu^+^, and Cu^2+^ surface sites. Furthermore, the Co and Cu cations can undergo reversible oxidation and reduction without disrupting the catalyst’s structure. While Co^2+^ sites activate H_2_O_2_ more efficiently than Cu^2+^ sites, the Co-Cu binary oxide catalysts exhibited superior NB removal performance compared to the Co_3_O_4_ catalyst, as shown in Figure 2, this phenomenon is effectively explained by Equation 6, which describes the interaction between Cu^2+^ and Co^3+^. Overall, based on the XPS analysis and quenching experiments, the H_2_O_2_ activation mechanism in the CuO-CoO_x_/SBA-15 system for NB degradation is depicted in Figure 7.

$${\text{Cu}}^{+}+H_{2}O_{2}\to {\text{Cu}}^{2+}+\cdot \text{OH}+{\text{OH}}^{-}$$ (1)

$$\cdot \text{OH}+H_{2}O_{2}\to H_{2}O+\cdot {\text{HO}}_{2}$$ (2)

$${\text{Cu}}^{2+}+H_{2}O_{2}\to {\text{Cu}}^{+}+2H^{+}+\cdot O_{2}^{-}$$ (3)

$${\text{Cu}}^{2+}+\cdot {\text{HO}}_{2}\to {\text{Cu}}^{+}+H^{+}+O_{2}$$ (4)

$${\text{Cu}}^{2+}+\cdot O_{2}^{-}\to {\text{Cu}}^{+}+O_{2}$$ (5)

$${\text{Cu}}^{+}+{\text{Co}}^{3+}\to {\text{Cu}}^{2+}+{\text{Co}}^{2+}$$ (6)

$${\text{Co}}^{2+}+H_{2}O_{2}\to {\text{Co}}^{3+}+{\text{OH}}^{-}+\cdot \text{OH}$$ (7)

$${\text{Co}}^{3+}+H_{2}O_{2}\to {\text{Co}}^{2+}+2H^{+}+\cdot O_{2}^{-}$$ (8)

$${\text{Co}}^{3+}+\cdot {\text{HO}}_{2}\to {\text{Co}}^{2+}+H^{+}+O_{2}$$ (9)

$${\text{Co}}^{3+}+\cdot O_{2}^{-}\to {\text{Co}}^{2+}+O_{2}$$ (10)

### 3.7 Catalyst reuse and recyclability

**FIGURE 8:**
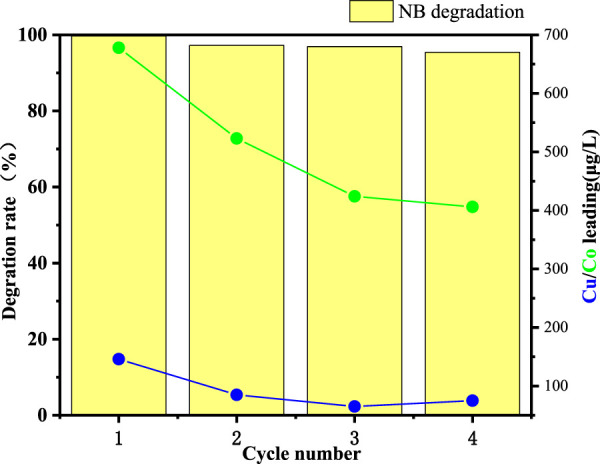
NB degradation performance of CuO-CoO_x_/SBA-15 and corresponding leached concentrations of Cu and Co across four reaction cycles.

The successful real-world application of heterogeneous Fenton-like degradation catalysts relies on their good reusability. In this study, the CuO-CoO_x_/SBA-15 catalyst was recovered from the precipitate at the bottom of the reaction solution after NB degradation and reused. As shown in Figure 8, CuO-CoO_x_/SBA-15 exhibited stable NB degradation performance over four consecutive reaction cycles under the same conditions. To assess the catalyst’s stability, ICP-MS was used to monitor the potential dissolution of metal ions in the recycled reaction solution after the 60-min reaction. After four cycles leached Cu and Co concentrations were found to be 0.075 mg/L and 0.407 mg/L, respectively, which comply with the World Health Organization (WHO) drinking water quality standards and the Chinese standard GB 25467-2010. These results confirm the excellent stability of the CuO-CoO_x_/SBA-15 catalyst, suggesting its protential for long-term use and highlighting the effectiveness of ultrasonic impregnation in encapsulating active metal components within the SBA-15 channels.

## 4 Conclusion

Mesoporous SBA-15 was synthesized using sol-gel and hydrothermal methods, and CuO-CoO_x_ nanoparticles were introduced into the mesopores of SBA-15 through ultrasonic impregnation. The resulting CuO-CoO_x_/SBA-15 catalysts exhibited highly porosity, large specific surface areas, and uniform dispersion of the active components.

HRTEM analysis revealed the uniformity of the Cu/Co bimetallic oxide particles and their excellent dispersion within the nanochannels of SBA-15. XPS analysis confirmed that H_2_O_2_ was activated by the Cu^+^ and Co^2+^ active sites on the CuO-CoO_x_/SBA-15 catalyst surface, with these active sites being oxidized to Cu^2+^ and Co^3+^. An efficient redox cycle between Cu^+^/Cu^2+^ and Co^2+^/Co^3+^ facilitated the continuous activation of H_2_O_2_.

The optimum CuO-CoO_x_/SBA-15 catalyst was prepared with a CuO-CoO_x_ content of 4.9 wt% and a Co/Cu molar ratio of 1:2. This catalyst demonstrated excellent NB removal performance over four reaction cycles, indicating good recyclability and stability. The optimal operating conditions for NB degradation in the CuO-CoO_x_/SBA-15-H_2_O_2_ system were an initial pH of 7–9, a CuO-CoO_x_/SBA-15 catalyst dosage of 2.0 g/L, and a H_2_O_2_ dosage of 200 mg/L. Under these conditions, 99.0% NB removal was achieved. The degradation of NB primarily proceeded via reaction with ·OH. Overall, the outstanding performance and stability of the CuO-CoO_x_/SBA-15 catalyst highlight its significant potential for practical treatment of refractory organic pollutants.

## Data Availability

The original contributions presented in the study are included in the article/Supplementary Material, further inquiries can be directed to the corresponding authors.
