# Supplementary material for: Heterogeneous Fenton-like CuO-CoOx/SBA-15 catalyst for organic pollutant degradation: synthesis, performance, and mechanism
Source: Front Chem. 2025 Feb 19;13:1552002. doi: 10.3389/fchem.2025.1552002 (PMC11880010; doi:10.3389/fchem.2025.1552002)
Supplement: Supplementary file 1 [file DataSheet1.pdf]

Supplementary Data
(5 pages)

Heterogeneous Fenton-like CuO-CoO$_x$/SBA-15 Catalyst for Organic Pollutant Degradation: Synthesis, Performance, and Mechanism

Jinwei Li[a], Yifei Wei[b], Qiang Liu[b],Huanhuan Guan[b], Chengchun Jiang[a*],Xiaohui Sun [c*]

(a. School of Material and Environmental Engineering, Shenzhen Polytechnic University, Shenzhen Guangdong 518055, China;
b. School of Municipal and Environmental Engineering,Shenyang Jianzhu University, Shenyang Liaoning 110168, China.)
c. College of Civil and Transportation Engineering, Shenzhen University, 3688 Nanhai Avenue, Shenzhen 518060, China.)

Corresponding Author: Chengchun Jiang
Tel: +86-755-26018380
Fax: +86-755-26018247
Email: jiangcc_szpt@126.com

Co-corresponding Author: Xiaohui Sun
Tel: +86-755-86670391
Fax: +86- 755-86670367
Email: sunxiaohui@szu.edu.cn

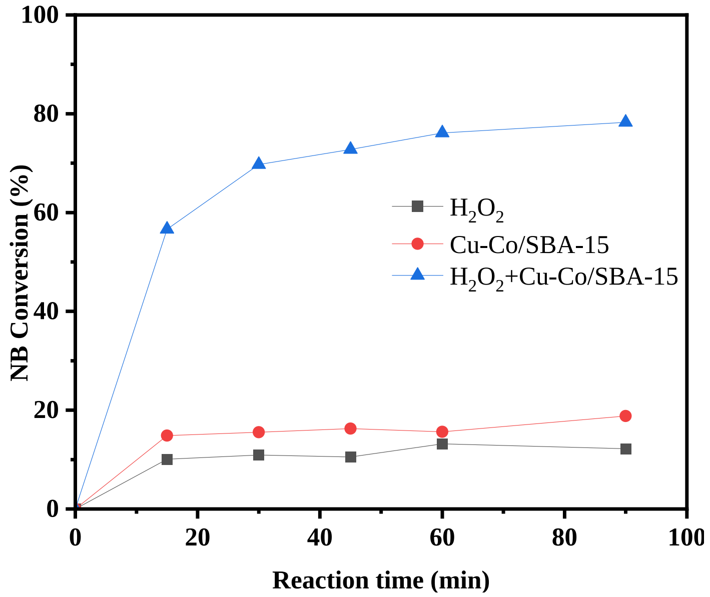

Figure S1. NB removal performance of H_2O_2 alone, CuO-CoO_x/SBA-15 catalyst alone, and CuO-CoO_x/SBA-15-H_2O_2 systems. Reaction conditions: 0.5 mmol/L NB, 100 mmol/L H_2O_2, and 2 g/L CuO-CoO_x/SBA-15.

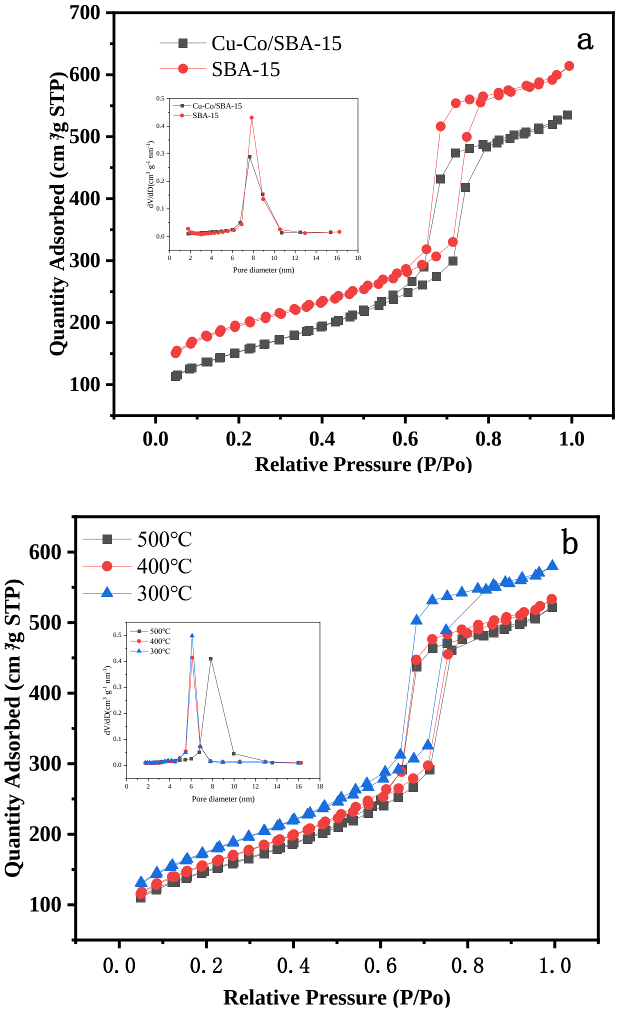

Figure S2. N_2 adsorption-desorption isotherms and pore diameter distributions of SBA-15 (a) and CuO-CoO_x/SBA-15 catalysts prepared using different calcination temperatures (b).

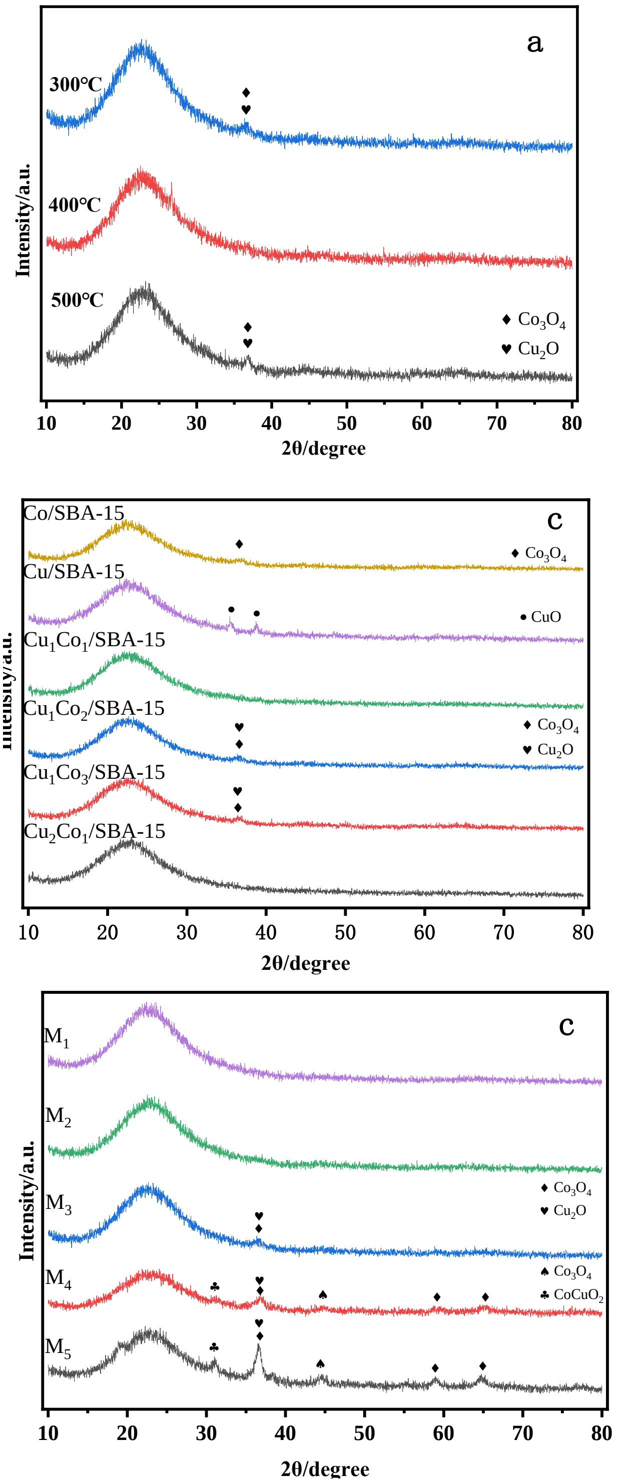

Figure S3. XRD patterns of CuO-CoO$_x$/SBA-15 catalysts prepared using different calcination temperatures (a), Cu/Co molar ratios (b), and metal oxide loading contents (c).

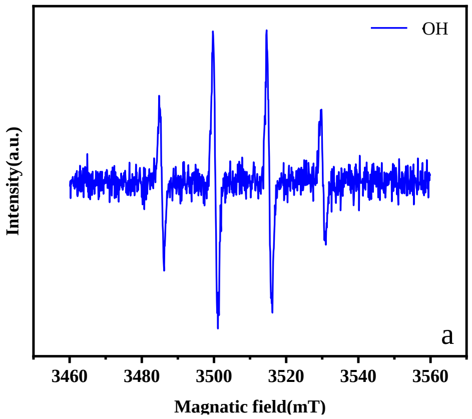

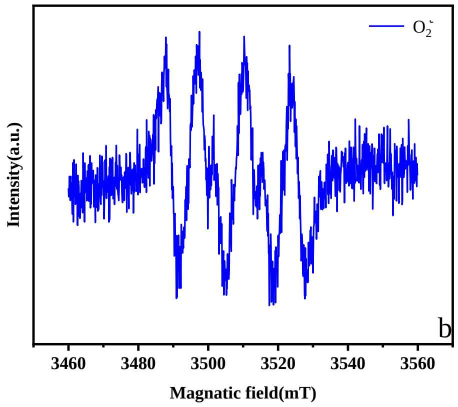

Figure S4. EPR spectra of CuO-CoO$_x$/SBA-15-H_2O_2 nitrobenzene system.

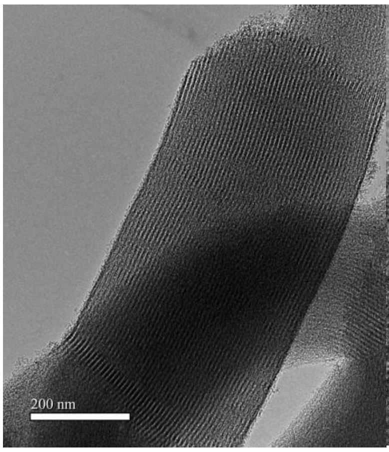

a TEM image of SBA-15

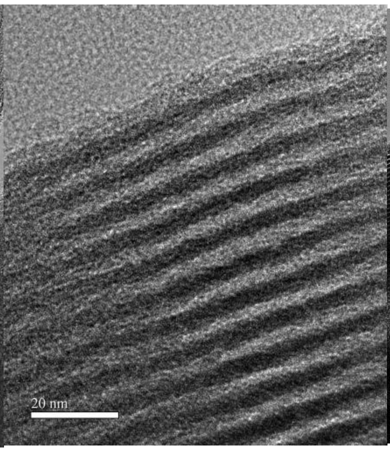

b TEM image of SBA-15

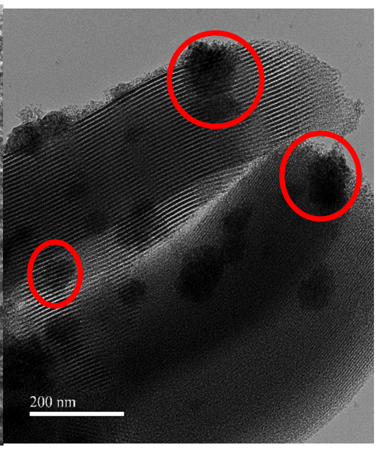

c TEM image of CuO-CoOx/SBA-15

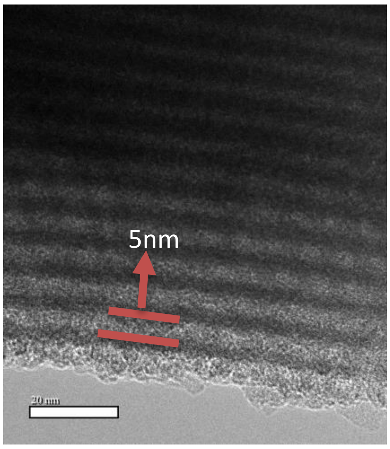

d TEM image of CuO-CoOx/SBA-15

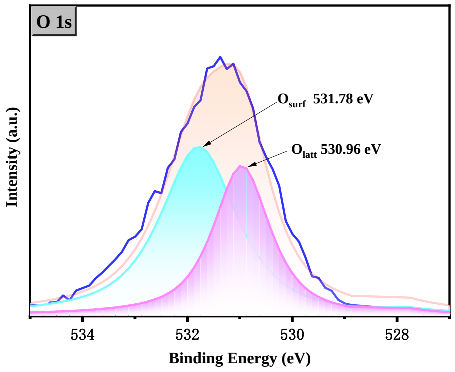

e O1s XPS spectra of CuO-CoOx/SBA-15

Figure S5. TEM images of SBA-15 (a,b) ,CuO-CoO$_x$/SBA-15 (c,d) and O1s XPS spectra of CuO-CoOx/SBA-15;
